# Randomized, Cross over, Multicenter, Single-Blind Study Comparing Citicoline 500 mg/Homotaurine 50 mg/Vitamin B3 54 mg/Pyrroloquinoline Quinone 5 mg (Neuprozin Mito^®^) and Citicoline 800 mg (Cebrolux^®^) on Pattern Electroretinogram (PERG) and Quality of Life in Patients with Primary Open-Angle Glaucoma with Well-Controlled Intraocular Pressure

**DOI:** 10.3390/jcm14113774

**Published:** 2025-05-28

**Authors:** Gemma Caterina Maria Rossi, Michele Rinaldi, Francesco Matarazzo, Diego Strianese, Giuseppe Campagna, Michele La Ragione, Paolo Esposito Veneruso, Giovanni Scapagnini, Ciro Costagliola

**Affiliations:** 1 Department of Surgical Sciences, University Eye Clinic, Fondazione IRCCS Policlinico S. Matteo, 27100 Pavia, Italy; gemma.rossi.md@gmail.com; 2 Department of Neurosciences, Reproductive Sciences and Dentistry, University of Naples “Federico II”, 80131 Naples, Italy; michele.rinaldi2@unina.it (M.R.); strianes@unina.it (D.S.); p.espositoveneruso@gmail.com (P.E.V.); ciro.costagliola@unina.it (C.C.); 3 Department of Physics “Ettore Pancini”, University of Naples “Federico II”, 80131 Naples, Italy; 4 Department of Medical-Surgical Sciences and Translational Medicine, University of Rome “Sapienza”, 00185 Roma, Italy; gius.campagna@gmail.com; 5 Department of Medicine and Health Sciences “V. Tiberio”, University of Molise, 86100 Campobasso, Italy; giovanni.scapagnini@unimol.it

**Keywords:** glaucoma, citicoline, homotaurine, pyrroloquinoline quinone, pattern electroretinogram, neuroprotection, quality of life, visual field

## Abstract

**Background/Objectives:** To evaluate the neuromodulative effects of oral intake of a fixed combination of citicoline 500 mg plus homotaurine 50 mg plus vitamin B3 54 mg plus pyrroloquinoline quinone (CIT/HOMO/B3/PPQ) or of citicoline 800 mg alone (CIT800) on retinal ganglion cell (RGC) function in glaucoma patients by pattern electroretinogram (PERG) and to investigate the effects on quality of life and visual function. **Methods:** Consecutive patients with primary open-angle glaucoma with controlled IOP (<18 mmHg) receiving prostaglandin analogues as monotherapy; with two reliable visual fields (Humphrey 24-2 SITA Standard) per year in the last 2 years; and an early to moderate visual field defect (MD < −12 dB) were randomized to: arm A. topical therapy + CIT/HOMO/B3/PPQ for 4 months, followed by 4 months of topical therapy + CIT800; and arm B. topical therapy + CIT800 for 4 months, then topical therapy + CIT/HOMO/B3/PPG for 4 months. Patients were examined at month 0, 4, and 8. Complete ocular examination, visual field test, PERG, and quality of life assessment (NEI-VFQ25) were performed at each visit. **Results:** Forty patients were selected and completed the study, and none developed or reported an adverse event. The overall mean age was 64.2 (±7.7) years, 27 were male. At the end of the intake period of both products, patients exhibited higher P50 and N95-wave amplitudes and shorter latencies compared to baseline. The crossover analysis found that PERG parameters were better when patients received the CIT/HOMO/B3/PQQ combination with a statistically significant shorter peak time of 1.24 ms (95% CI, 0.37 to 2.10; *p* = 0.006) in the central P50 wave, 1.32 ms (95% CI, 0.44 to 2.22; *p* = 0.004) in the inferior P50 wave, and 1.70 ms (95% CI, 0.09 to 3.31; *p* = 0.038) in the inferior N95 wave; and a statistically significant increase of 0.35 µV (95% CI, 0.10 to 0.60; *p* = 0.006) in the superior N95 amplitude. The crossover analysis did not reveal any significant differences between the intake of CIT800 and CIT/HOMO/B3/PQQ in terms of visual acuity or IOP. During the intake of CIT/HOMO/B3/PQQ, a significant improvement was observed in the total mean score (*p* = 0.004), in the general health scale (GH, *p* = 0.01), in the color vision scale (*p* = 0.006), and in the peripheral vision scale (*p* = 0.001). **Conclusions:** The present study has shown that the addition of CIT/HOMO/B3/PQQ in early glaucoma improves PERG parameters and quality of life, likely by slowing down RGC aging and enhancing mitochondrial function more significantly than citicoline 800 mg alone.

## 1. Introduction

Glaucoma is a progressive and multifactorial neurodegenerative disorder involving retinal ganglion cells (RGCs) and their axons. Currently, the only approach proven to be effective in preserving visual function is lowering intraocular pressure (IOP), both in the initial and advanced stages [1,2,3,4]. However, in some cases, the disease progresses despite adequate intraocular pressure control, indicating that glaucoma is a complex multifactorial condition involving IOP-independent neurodegenerative mechanisms. This causes trans-neuronal degeneration involving the eye, the optic nerve, and the brain [5]. For this reason, glaucoma is now considered a neurodegenerative process [6,7,8] characterized by neuron loss, oxidative stress, apoptosis, excitotoxicity, abnormal protein accumulation, and trans-synaptic degeneration, which leads to anterograde trans-synaptic neurodegeneration triggered by retinal ganglion cell death, and retrograde trans-synaptic neurodegeneration, induced by neurodegenerative processes in the central nervous system [6].

These new observations have encouraged the exploration of treatment modalities beyond IOP control, emphasizing neuroprotective and neuromodulatory agents. In recent years, research has focused on finding neuroprotective agents to complement standard IOP-lowering therapy, and major glaucoma societies have begun to consider this new therapeutic option.

In 2021, the European Glaucoma Society guidelines [9] defined neuroprotection as “a therapeutic approach aiming to directly prevent, hinder, and, in some cases, reverse neuronal cell damage”, recognizing the potential role of neuromodulating agents.

Experimental models of glaucoma have shown that retinal ganglion cells die through apoptosis. The fate of RGCs is determined by a cross-talk between pro-apoptotic signals and survival promoters. Several compounds have been shown to be neuroprotective in animal models of experimental glaucoma. So far, several studies on citicoline have demonstrated its neuroprotective properties. In particular, glaucoma patients with “controlled IOP” treated with an oral citicoline solution (500 mg/day) for 4 months, divided by 2-month no-treatment periods, showed a reduction in the rate of progression of visual field defects [10].

Other studies have demonstrated that citicoline acts as a neuroenhancer both in the central nervous system and in the eye ag high doses (1000 or 1600 mg/day) [11,12,13,14,15].

Homotaurine has been shown to inhibit the formation of β-amyloid plaques [16], which are responsible for several neurodegenerative diseases within the central nervous system and in the ophthalmic field [17]. Some studies on retinal mixed cells of Wistar rats have shown a synergistic action of citicoline and homotaurine in reducing cytotoxicity and apoptosis in a glaucoma model, and two previous clinical studies supported these observations [18,19].

Vitamin B3, also noted as nicotinamide, is an essential molecule in energy and redox metabolism. Its levels decline with age, and previous studies have shown that it is significantly reduced in several neurodegenerative disorders, including glaucoma, thereby contributing to neuronal dysfunction and disease-related aging processes [20].

In 2017, Williams et al. [21] demonstrated that the administration of high doses of vitamin B3 prevents the development of glaucoma in an experimental model. This observation opened the door to several studies on this molecule in clinical/human settings. A recent review [22] reported that the literature has shown a strong connection between retinal ganglion cell (RGC) dysfunction and reduced nicotinamide levels. While the majority of evidence derives from animal models, a limited number of studies have also been conducted in humans; however, an optimal dosing regimen has yet to be established. However, a pilot clinical trial has recorded an improvement in inner retinal function in glaucoma with a daily intake of 3 g [23].

Pyrroloquinoline quinone (PQQ) is associated with biological processes such as mitochondriogenesis, reproduction, growth, and aging. In human clinical trials, PQQ was reported to promote cognitive function and improve regional blood flow in older adults [24]. As mitochondria play an important role in the pathogenesis of various ophthalmic diseases (e.g., glaucoma), multiple strategies targeting mitochondrial protection against oxidative stress and premature apoptosis should be devised to protect RGCs [25].

Glaucoma is a complex disease that requires intervention in multiple degenerative pathways. The literature has demonstrated a significant neuromodulator role of citicoline, and some studies have recorded additional positive neuroprotective effects when it is combined with other neuromodulators [19]. The general aim of this study was to evaluate the potential beneficial effects of supplementation with a fixed combination of 500 mg citicoline, 50 mg homotaurine, 54 mg of vitamin B3, and pyrroloquinoline quinone on RGC function in subjects with glaucoma, as measured by pattern electroretinogram, in comparison to 800 mg citicoline alone. Based on a PubMed search, this is the first study to provide a direct head-to-head comparison between citicoline and a combination of different neuromodulating molecules, examining their effects on function and quality of life.

## 2. Methods

**Figure 1:**
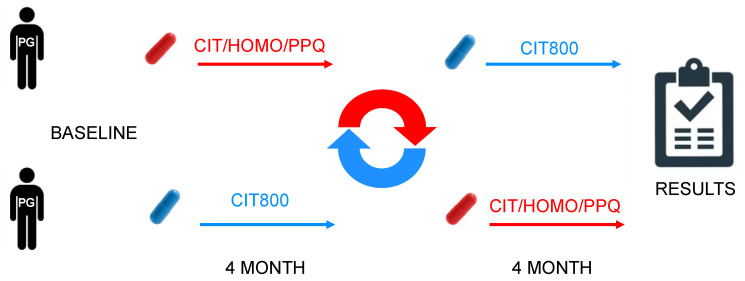
Design cross over.

A multicenter, randomized, cross-over, single-blind study was conducted (Figure 1). The cross-over design was implemented to limit intraindividual variability. Blinding was not considered necessary, as the patient could not interfere with the measurement of the pattern-electroretinogram (PERG).

**Figure 2:**
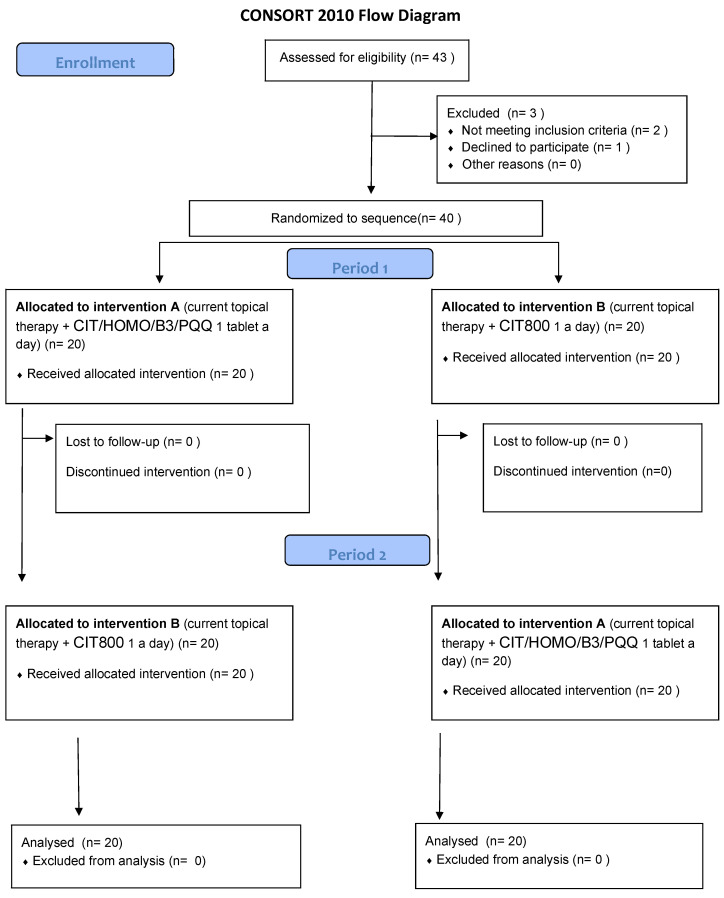
CONSORT 2010 NPFlow.

The study was conducted in accordance with the recommendations of the Declaration of Helsinki (revision 2000, Edinburgh) and the Italian legislation on Good Clinical Practice (Ministerial Decree of 15 July 1997 and subsequent amendments). It was approved by the local Ethics Committee (“Comitato Etico Campania 3” Prot. N. 00002505, 23 January 2024). The study complied with CONSORT 2010 guidelines (Figure 2) and was registered on ClinicalTrials.gov (NCT identifier NCT06431113).

All eligible patients were asked to complete the study after signing the informed consent form. The study was not supported by any industry and did not receive any form of financial grant or sponsorship. At the request of the Ethics Committee, FB Vision (the Italian company that produces the CIT/HOMO/B3/PQQ fixed combination) provided all product packages (both CIT/HOMO/B3/PQQ and CIT800) free of charge for all patients throughout the entire duration of the study. Subjects who withdrew consent or who, based on the clinician’s professional assessment, were no longer suitable for participation were considered withdrawn.

Patients were selected according to the eligibility criteria summarized in Table 1.

### 2.1. Primary Outcome

To compare the effects on PERG of one tablet per day of the fixed combination of citicoline 500 mg, homotaurine 50 mg, vitamin B3 54 mg, and Pyrroloquinoline Quinone 5 mg (Neuprozin Mito^®^—CIT/HOMO/B3/PQQ, FB-Vision, San Benedetto del Tronto AP, Italy) versus one tablet of citicoline 800 mg (Cebrolux^®^—CIT800, Bausch & Lomb, New York, NY, USA), as an add-on to standard topical therapy.

### 2.2. Secondary Outcomes

To compare the two treatments over time in terms of visual acuity, visual field changes, quality of life perception (NEI VFQ25 questionnaire), and safety (incidence of adverse events).

### 2.3. Study Treatments

#### 2.3.1. Standard Treatment Currently Available in Clinical Practice

The standard treatment for glaucoma consists of the administration of eye drops that reduce intraocular pressure, with prostaglandin derivatives being the most effective agents [7]. As defined in the eligibility criteria, selected patients must have been on topical therapy with prostaglandin derivatives alone, once daily (bimatoprost, latanoprost, travoprost, tafluprost) for at least one year.

#### 2.3.2. Investigational Treatment

Patients were randomized into two arms:

Arm A: CIT/HOMO/B3/PQQ supplementation (from randomization to month 4), followed by CIT800 (from month 4 to month 8), in addition to standard topical treatment.

Arm B: CIT800 supplementation (from randomization to month 4), followed by CIT/HOMO/B3/PQQ (from month 4 to month 8), in addition to standard topical treatment. Both tablets were prescribed at 8:00 AM.

#### 2.3.3. Planned Assessments

At each visit, all patients underwent a complete ophthalmic examination, which included visual acuity (VA), anterior segment evaluation (anterior chamber, lens, and angle), IOP measurement (Goldmann tonometry), and optic nerve evaluation with an indirect lens (Volk 90). All patients also underwent pattern electroretinogram (PERG) and visual field examination (Humphrey 24-2 SITA-Standard). Additionally, all patients completed the Italian version of the NEI VFQ-25 questionnaire to assess their quality of life.

Other anamnestic and demographic variables were collected for each subject at baseline and over time, including date of birth (month/year), gender, therapy (systemic and topical), medical history (systemic and ocular, including previous lasers/surgeries), and ocular parameters (lens, optic nerve, and retina).

### 2.4. Study Definitions and Diagnostic Criteria

#### 2.4.1. Glaucoma Diagnosis

The diagnosis of glaucoma required: IOP > 21 mmHg on at least two consecutive visits at the time of first diagnosis, the presence of glaucomatous optic nerve head (ONH) damage confirmed by an expert fundus examination, and at least three reliable Humphrey 24-2 full-threshold visual field tests performed on different days showing a glaucomatous or suspected glaucomatous defect.

#### 2.4.2. Ocular Examination

Visual acuity was determined in decimal units using Snellen charts. Slit-lamp examination of the anterior and posterior segments was conducted with particular attention to the retina and optic nerve. IOP was measured using Goldmann tonometry, and the ophthalmologist performing the measurement was blinded to study procedures.

#### 2.4.3. Visual Field Examination

The Humphrey Visual Field is an automated procedure used to perform perimetry, a test that measures the entire area of peripheral vision while the eye is focused on a central point. During this test, lights of varying intensities appear in different parts of the visual field while the patient’s eye is focused on a fixed spot. The perception of these lights is charted and compared to the results of a healthy eye of the same age to determine if any damage has occurred. This procedure is quick and effective, typically completed in about 10 min, and is used to diagnose and monitor the progress of glaucoma.

Main parameters used to evaluate damage and progression include:

Mean Defect and Pattern Standard Deviation (MD and PSD, measured in decibels).

Glaucoma Hemifield Test (GHT), a qualitative description of the examination, classified as normal, borderline, or outside normal limits.

Visual Field Index (VFI), a global index that assigns a number between 1% and 100% based on an aggregate percentage of visual function, with 100% representing a perfect age-adjusted visual field.

All patients underwent the 24-2 SITA-Standard Humphrey Visual Field examination.

### 2.5. Electrofunctional Examination

#### 2.5.1. Pattern Electroretinogram (PERG)

Electroretinography (ERG) measures the retinal bioelectrical response to a visual stimulus [26,27]. The stimulus can either be a flash of stroboscopic light (referred to as flash ERG) or a pattern structured on a television monitor, in which black and white bars or checkerboards alternate over time (referred to as PERG). ERG reflects the functionality of the outermost retinal layers (pigmented epithelium and photoreceptors), while the PERG is generated by the innermost retinal layers (ganglion cells).

In PERG, a series of peaks can be identified, each marked with a letter indicating polarity (P for positive, N for negative) and a number indicating latency time and amplitude of hte wave.

Latency time in PERG refers to the time interval, measured in milliseconds (ms), between the presentation of the visual stimulus and the appearance of each peak in the waveform. The key latency times associated with PERG are N35, P50, and N95, with N35 typically occurring around 35 ms, P50 around 50 ms, and N95 around 95 ms. Delayed latency times can indicate dysfunction in the retinal ganglion cells or their pathways.

The amplitude of the PERG waveform refers to the height of the peaks, typically measured in microvolts (µV), and reflects the strength of the retinal ganglion cell response to the visual stimulus. The main amplitudes in PERG are the P50 and the N95. Higher amplitudes generally indicate better retinal function.

PERG is an objective and direct measure of inner retinal function correlated with retinal ganglion cell (RGC) activity. It has been shown that PERG can document and predict early glaucomatous changes [26,27].

The PERG is a retinal biopotential evoked when a temporally modulated patterned stimulus (checkerboard or grating) of constant mean luminance is viewed. PERG is a very small signal, typically in the range of 0.5–8 µV, depending on the characteristics of the stimulus. As a result, PERG recording is technically more demanding than conventional ERG.

PERG was recorded according to the International Society for Clinical Electrophysiology of Vision guidelines using Retimax^®^, a glaucoma Hemifield PERG test (CSO, Florence, Italy). This device features a large 55″ screen and hardware that eliminates luminance influence on PERG, repeating the stimulation twice in each session using a specific software (hemifield stimulation) that allows for intra-individual variability assessment. The test records the wave in the center, as well as in the superior and inferior hemifields.

#### 2.5.2. NEI VFQ-25 Item

The patients’ quality of life was assessed using the Italian version of the 25-item National Eye Institute Visual Function Questionnaire [28]. The 25-item NEI-VFQ is a vision-targeted, non-disease-specific instrument designed to measure the impact of ocular disorders on vision-related quality of life. Depending on the item, responses to the questionnaire pertain to the frequency or severity of a symptom or problem with functioning. NEI-VFQ scores can range from 0 to 100, with lower scores indicating more problems or symptoms.

#### 2.5.3. Single-Blind Procedures

All personnel involved in performing the visual field examination, optic nerve evaluation, IOP measurement, and electrophysiological examinations were blinded to the patient’s treatment period. The data analyst was also blinded to the treatment group.

#### 2.5.4. Adverse Events

Systemic and topical adverse events (AEs) were collected at each visit. Patients were asked about the comfort of the therapy and any side effects.

For each AE, the following information was recorded in the patient’s medical chart: nature of the adverse event, date and time of occurrence and disappearance (i.e., duration); intensity: mild, moderate, or severe; frequency: once, continuous, or intermittent; decision regarding study participation: continuation or withdrawal; relationship to the study medication; measures taken to treat it; AEs were treated according to standard clinical practice. In the event of a serious adverse event, the drug was discontinued.

#### 2.5.5. Primary Endpoint

To compare the effects of adding the fixed combination of citicoline 500 mg plus Homotaurine 50 mg plus pyrroloquinoline quinone (Neuprozin Mito^®^—NPM) a tablet a day on PERG examination (p50 wave) at four months of therapy, compared to citicoline 800 mg alone, as an add-on to standard topical therapy.

### 2.6. Elements for Sample Size Calculations or Study Power

#### 2.6.1. Sample Size

This study was designed as a cross-over where half of the patients will be assigned to sequence AB, i.e., A: CIT/HMO/B3/PQQ and B: CIT800 receiving A in the first period of treatment, no washout, and then B in the second treatment period. The other half will be assigned to the BA sequence, following the same schedule but with reversed supplements. In each treatment period, the value of the latency time of the PERG P50 will be evaluated in order to determine the sample size. These values, for the calculation of the sample size, have been identified in the articles of Parisi V. et al. (1999) [11] and Rossi GCM et al. (2022) [19]. Following the approach of Senn S. and the formulas of Steven A.J. (1993 chapter 3 and 2010 formulas 3.3 and 4.5) [29,30], in which we assume the absence of period effect, carry-over, and each pair of measurements on the same subject will have a correlation of approximately r = 0.3 (conservative), we obtain a standard deviation (σ_diff_ = 7.92). Having established a clinically reliable difference (MCID: Minimal Clinically Important Differences) between 6 ≤ d ≤ 8, we obtain with a power of 80% and a cut-off of α = 0.05 a number of n = 32 patients. Considering a drop-out of 20%, a total of n = 40 patients will be enrolled, rounded up. This result is obtained by applying the proc power pairedmeans test = diff performed using the SAS v. 9.4 TS Level 1M8 (SAS Institute, Cary, NC, USA).

#### 2.6.2. Analysis Plan

Categorical variables will be expressed as absolute and percentage frequencies—n (%); while continuous variables will be shown as mean ± standard deviation and 95% Confidence Interval.

The comparison at baseline between the two groups A: Citicoline 800 mg + Homotaurine 50 mg + vitamin B3 54 mg + PQQ 5 mg (CIT/HOMO/B3/PQQ) and B: Citicoline 800 mg (CIT800) of the continuous variables will be evaluated by Student’s t (verified normality) or Brunner–Munzel (normality violated). The normality of the continuous variables will be tested by Shapiro–Wilk test and checking of the Q-Q plot; while the association between the two groups and the categorical variables will be evaluated either with the X^2^ test or Fisher’s exact test when necessary.

The cross-over analysis will be performed through a mixed model (Grizzle model) belonging to the family of generalized linear models. This model allows us to evaluate the differences between group A and group B and the possible effect of the carry-over (absent when *p* > 0.05). Statistical analysis will be performed using SAS v. 9.4 TS Level 1M8 and/or JMP PRO v. 17 (SAS Institute, Cary, NC, USA).

A probability of *p* < 0.05 will be considered statistically detectable.

## 3. Results

Forty patients were enrolled and signed informed consent; all patients completed the clinical trial, and none developed or reported an adverse event. All enrolled patients were Caucasian and diagnosed with primary open-angle glaucoma (POAG). Demographic and anamnestic data are presented in Table 2.

The overall mean age was 64.2 ± 7.7 years, with 27 patients (67.5%) being male. Most patients had one or more systemic diseases. In particular, 16 patients (57%) had systemic hypertension, 12 (43%) had hypercholesterolemia, eight (28%) had vascular disorders (e.g., acute myocardial infarction, cerebral infarction, cardiomyopathy, atrial fibrillation), and five (18%) had diabetes.

All patients were on prostaglandin derivative monotherapy, as per the inclusion criteria, with the majority using Tafluprost (17 patients, 42%) and Latanoprost (12 patients, 30%). All patients had an early glaucomatous visual field defect, and visual acuity was very good. Twenty percent of patients were pseudophakic, and intraocular pressure was well controlled. Table 2 summarizes all data at baseline, per eye.

Baseline demographic and clinical characteristics were well-balanced between the two arms, with no statistically significant differences

### 3.1. Electrophysiology

Regarding the transient PERG examination, at the end of the intake period (either of CIT800 or CIT/HOMO/B3/PQQ), patients exhibited higher P50 and N95-wave amplitudes and shorter latencies compared to baseline, across all examined fields (central, superior, and inferior). This improvement was statistically significant for nearly all examinations (Table 3). More significant improvements were observed during the intake of the CIT/HOMO/B3/PQQ combination, with better results recorded in the inferior and superior sectors (Table 3).

The crossover analysis, summarized in Table 4, revealed that almost all electrophysiological parameters were better when patients received the CIT/HOMO/B3/PQQ combination. Specifically, the CIT/HOMO/B3/PQQ combination resulted in a statistically significant shorter peak time of 1.24 ms (95% CI, 0.37 to 2.10; *p* = 0.006) in the central P50 wave, 1.32 ms (95% CI, 0.44 to 2.22; *p* = 0.004) in the inferior P50 wave, and 1.70 ms (95% CI, 0.09 to 3.31; *p* = 0.038) in the inferior N95 wave. A statistically significant increase of 0.35 µV (95% CI, 0.10 to 0.60; *p* = 0.006) was recorded in the superior N95 amplitude. These results were independent of sequence, age, gender, visual field defect, IOP values, and type of prostaglandin used.

### 3.2. Visual Acuity, Visual Field, and Intraocular Pressure

Visual acuity and visual field parameters, including both mean deviation (MD) and pattern standard deviation (PSD), significantly improved at the end of the intake period of CIT/HOMO/B3/PQQ (Table 5).

The crossover analysis did not reveal any statistically significant differences between the intake of CIT800 and CIT/HOMO/B3/PQQ in terms of visual acuity or intraocular pressure (IOP).

However, the crossover sequence analysis showed a significant improvement in visual acuity with the sequence NeuprozinMito^®^/Cebrolux^®^ (Table 6). Additionally, while the crossover analysis did not find statistically significant differences in MD or PSD due to the intake of CIT800 or CIT/HOMO/B3/PQQ, the sequence analysis revealed a significant improvement in PSD with the Cebrolux^®^/NeuprozinMito^®^ sequence (Table 6).

### 3.3. Quality of Life

Quality of life scores before and at the end of the intake period of both therapies are collected in Table 7. Table 8 shows the results of the crossover analysis regarding quality of life, as assessed using the NEI VFQ-25. During the intake of CIT/HOMO/B3/PQQ, a significant improvement was observed in the total mean score (*p* = 0.004), in the general health scale (GH, *p* = 0.01), in the color vision scale (*p* = 0.006), and in the peripheral vision scale (*p* = 0.001).

## 4. Discussion

The present study found that the parameters of the pattern electroretinogram (PERG) generally improved after four months of daily intake of either CIT800 or CIT/HOMO/B3/PQQ, when added to monotherapy with prostaglandin derivatives. The crossover analysis revealed that almost all electrophysiological parameters improved when patients took the CIT/HOMO/B3/PQQ combination, with significant effects observed in the central and inferior P50 wave latency, inferior N95 wave latency, and superior N95 amplitude. Additionally, quality of life, as assessed using the NEI-VFQ25, significantly improved during the intake of CIT/HOMO/B3/PQQ, with notable improvements in the total mean score and in specific scales, particularly the general health scale, color vision scale, and peripheral vision scale. Glaucoma is a group of eye conditions that cause damage to the optic nerve, which transmits visual information from the eye to the brain. This relationship has become increasingly important in recent years, and the understanding that glaucoma is not only a disease of the eye has grown, now being widely recognized by the scientific community [5,6].

It is equally clear that while intraocular pressure (IOP) is the primary risk factor for developing glaucoma and the cornerstone of glaucoma therapy, it is not always the only target of treatment. In fact, damage to the optic nerve, due to the loss of function of retinal ganglion cells (RGCs), is often a result of increased intraocular pressure, but RGCs may also degenerate for other reasons, such as oxidative stress, inflammation, and excitotoxicity [6].

Neuroprotection in glaucoma focuses on preventing or slowing the degenerative process of RGCs, in addition to IOP control. Based on these considerations, it is understandable that acting on multiple fronts is necessary to achieve better neuroprotective outcomes.

IOP reduction itself is considered an “indirect neuroprotection”, as reducing the pressure within the eye helps alleviate mechanical stress on the optic nerve, thereby preventing further damage [31]. Beyond merely lowering IOP, neuroprotective strategies aim to protect retinal ganglion cells and other neurons in the optic nerve from the various stressors mentioned earlier.

The present study examined stable patients with well-controlled intraocular pressure (IOP) and early defects to avoid the indirect neuroprotective effect of IOP lowering, focusing on the neuromodulatory effects of a newly available fixed combination of the most widely studied neuroprotective agents, compared to citicoline alone, which is the most studied agent and has the most available evidence. This study was challenging, as there are no studies to date comparing the effects of citicoline alone versus the effects of multiple agents with different neuromodulatory targets of action.

As mentioned, some clinical studies have focused on evaluating promising agents. The most studied and widely published ones include neurotrophic factors (Nerve Growth Factor, Ciliary Neurotrophic Factor [CNTF]), antioxidants (Coenzyme Q10, nicotinamide, citicoline), and mitochondrial agents (nicotinamide, coenzyme Q10, Ginkgo biloba), among others [32]. Regarding the neurotrophic agents mentioned above, most have been explored for their potential to protect retinal ganglion cells in animal models of glaucoma. However, their clinical application is still under investigation, with the main challenge being the delivery of these agents to the retina in sufficient quantities and verifying their potential in a clinical setting.

Our study revealed a significant effect of the combination of multiple agents compared to citicoline alone. It is important to note that most actual studies on the effects of citicoline in glaucoma are based on a 500 mg dosage. In our study, we adopted an 800 mg dosage, which increased the challenge, as it has been previously demonstrated that better effects on retinal function and neural conduction in the visual pathways of glaucoma patients are obtained with higher dosages, similar to the approach used for other degenerative brain disorders [33].

Regarding the results we obtained, it should be noted that the present study was primarily conducted to assess improvements in both the PERG and the quality of life of patients.

PERG is a diagnostic test used to evaluate the function of the retina, specifically the retinal ganglion cells. It can help detect early signs of damage before it becomes detectable through other methods, such as visual field testing (the gold standard for diagnosing and monitoring glaucoma) or optic nerve evaluation. Therefore, it is particularly useful in early-stage glaucoma. However, it is also a valuable tool for monitoring the progression of glaucoma over time and assessing the effects of neuromodulatory or neuroprotective agents.

Our results demonstrated a significant improvement in both the amplitudes and latencies of the P50 and N95 waves. Upon crossover analysis, the CIT/HOMO/B3/PQQ combination showed superiority over citicoline alone, particularly for the N95 wave.

PERG is a functional assessment, and several studies have demonstrated significant differences in most PERG parameters in glaucoma patients, which can be summarized by prolonged P50 and N95 latency and decreased P50 and N95 amplitude [34,35]. However, there is no agreement on which of the two waves is the most specific parameter to consider. Various results have been published: Demir et al. [36] found that N95 amplitude was lowered more than P50 amplitude in primary open-angle glaucoma (POAG); Jung et al. [37] showed that N95 amplitude is the most important parameter to assess; Ventura et al. [38] reported that ganglion cells generate the N95 wave, and by measuring its amplitude, one can infer the approximate number of ganglion cells; North et al. [39] showed that N95 amplitude discriminates between ocular hypertension and glaucoma; Elgohary et al. [40] found that P50 latency and N95 amplitude varied significantly between glaucoma suspects and POAG patients, whereas N95 latency varied significantly between normal and POAG; Cvenkel et al. [41] discovered that both the P50 and N95 amplitudes are sensitive for early glaucoma, but they did not include P50 or N95 latency in their study.

These differences may be due to varying patient selection based on disease severity and age, but they are also linked to the different study designs, some of which only analyzed specific PERG parameters and did not use the same equipment or examination program for the PERG. In general, however, there is scientific evidence that both waves are important and are altered in glaucoma patients. Even if, in early glaucoma, the N95 wave seems to be more specifically altered than the P50 wave [38,41].

One limitation of this study is the potential for self-selection bias, as participants voluntarily agreed to undergo supplementation and testing. Additionally, the study population was relatively homogeneous in terms of ethnicity, which may limit the generalizability of the findings to broader and more diverse populations.

Another limitation of the study could be the absence of a control group that did not receive either citicoline or the combination under investigation; however, previous literature has already demonstrated a positive effect on PERG of citicoline, either alone or in combination with homotaurine, compared to a control group not receiving these active compounds; therefore, it was not considered necessary to include a third group in the crossover design. Moreover, in relation to the potential risk of carryover and the absence of a washout period in the study design, we conducted a specific statistical analysis to evaluate possible residual effects between treatment phases. The results did not indicate any significant carryover effect.

While this study provides encouraging preliminary evidence, future studies should aim to include larger cohorts with greater ethnic diversity and longer follow-up periods to better assess the long-term effects of supplementation.

The patients we examined in the present study had an early visual field defect, which is why we wanted to mainly rely on the data published by Ventura, who found that in early glaucoma, the reduction in RGC electrical activity exceeds the proportion expected from lost RGC axons [38]. This indicates that, in patients with early-stage glaucoma, the total amount of RGC electrophysiological activity is, on average, less than expected based on the loss of RGC axons. The authors concluded that an undefined proportion of central RGCs is dysfunctional and may undergo premature death if the causes of dysfunction are not reduced or blocked. The magnitude of the disproportionate PERG amplitude loss is about 33% in early glaucoma.

The positive and effective neuromodulatory effects observed in our study after the intake of neuroprotective agents may be due to their ability to block or modulate the causes of dysfunction. The combination of multiple agents (CIT/HOMO/B3/PQQ) likely promotes greater efficacy in RGC electrical activity. We hypothesize that these effects are due to the synergistic action of the agents we used. Citicoline is well-known for its actions in membrane protection and repair, not only at the level of RGC membranes but also at the mitochondrial membrane level. Our data confirm these observations: in fact, PERG significantly improved during the intake of CIT800 alone. However, our study recorded a better and more significant effect on PERG with the combination of CIT/HOMO/B3/PQQ. This can likely be explained by the fact that PQQ and nicotinamide (B3) exert antioxidant effects primarily at the mitochondrial level, blocking or at least slowing down aging, while homotaurine stabilizes neuronal membranes, reduces oxidative stress, decreases excessive glutamate release, and modulates excitotoxicity (also reducing the accumulation of amyloidogenic proteins that interfere with cell survival).

Regarding quality of life, we know that in glaucoma, the first ganglion cells to fail are typically those located in the superior and inferior regions of the retina, and visual field loss generally proceeds from the peripheral to the central field. Our data on quality of life perception during the intake of CIT/HOMO/B3/PQQ recorded a significant improvement across different scales. However, what we would like to emphasize is the statistical improvement in the peripheral vision scale, which likely reflects the improvement observed in both the inferior and superior PERG waves and the reduction in RGC dysfunction present at baseline. These positive effects on quality of life support the hypothesis that the agents studied have neuroenhancing effects.

## 5. Conclusions

A natural decline in retinal ganglion cells occurs with aging, but this process is more pronounced and accelerated in conditions such as glaucoma. Neuroprotection aims to increase the energy of RGCs to stabilize them and prevent further dysfunction.

PERG is a tool that can discriminate between normal, glaucoma suspect, and early glaucoma patients, and is particularly useful in those presenting with early visual field defects. Our data show a positive effect on PERG waves, and therefore on RGC electrical activity, likely due to the recovery of dysfunctional cells, especially during the intake of a combination of agents that target cell aging, oxidative stress, and mitochondrial damage, such as the CIT/HOMO/B3/PQQ fixed combination, in comparison to citicoline alone.

A positive effect on quality of life was also observed, with particular improvement in the peripheral vision scale, reflecting a neuroenhancing effect.

In conclusion, the present study has shown that the addition of CIT/HOMO/B3/PQQ in early glaucoma improves PERG parameters and quality of life, likely by slowing down RGC aging and enhancing mitochondrial function more significantly than citicoline 800 mg alone.

## Figures and Tables

**Table 1:** Inclusion and exclusion criteria.

Inclusion Criteria	Exclusion Criteria
Age >18 years; Diagnosis of primary OAG (POAG) from, at least, 3 years; Visual acuity >0.7 (7/10) decimals; Refractive error <5 D (spheric) and <2D (cylinder); Transparent diopter means (cornea and lens); Controlled IOP (<18 mmhg, morning value) with prostaglandine analogues as monotherapy; Stable IOP < 18 mmhg in the last 2 years; Stable and unchanged topical therapy in the last 6 months; At least two reliable visual fields (humphrey 24-2 sita standard) per year in the last 2 years; Early visual field defect (md < −12 db); Electrophysiological (PERG) parameters alterations similar to glaucomatous pathology; Written consent to participate in study procedures and data utilization in an anonymous form	Normal tension glaucoma; History of recurrent uveitis/scleritis/herpes infection; Pregnancy and breastfeeding; Contraindication to citicoline and/or homotaurine and/or PQQ Contraindication prostaglandine analogues Topical therapy with Brimonidine or beta-blockers as monotherapy or fixed combination Topical therapy with pilocarpine and aceclidine, monotherapy, or fixed combination Systemic or topical treatment with another neuroprotective agent in the last 4 months prior to enrollment Systemic therapies affecting patients’ performance in visual field examination (sedatives); Glaucomatous scotomas within 10 degrees from fixation Any condition limiting the patient’s ability to participate in the study; Other ocular causes of visual field and PERG changes, such as cataract, myopic chorioretinopathy, macular diseases, retinal vascular occlusion, diabetic retinopathy; Other systemic causes of visual field and PERG changes such as neurodegenerative disorders (Alzheimer’s disease, Parkinson’s disease, ALS, MS) or pituitary disorders; Cerebral ischemia in the last 2 years; Any change in topical therapy in the 6 months prior to enrollment or during the study period; Concomitant participation to another clinical trial; Any previous filtering and/or retinal surgery; Cataract surgery in the last 6 months; Any previous laser treatment for glaucoma in the last 5 years.

**Table 2:** Demographic and clinical data at baseline.

Parameter		Parameter	Right Eye	Left Eye
	*n* (%)		Mean ± SD	Mean ± SD
**Gender**		**Visual field**		
Male	27 (67.5)	MD (db)	−2.39 ± 2.15	−2.63 ± 1.95
Female	13 (32.5)	PSD (db)	2.7 ± 1.3	3.2 ± 1.4
	Mean ± SD			
**Age** (years)	64.2 ± 7.7	**Visual acuity** (decimals)	9.15 ± 1.4	8.8 ± 2.1
**Years of topical therapy**	3.1 ± 2.3	**Intraocular pressure** (mmHg)	12.8 ± 2.9	12.9 ± 2.6
	N (%)			
**Systemic pathologies**		**Central PERG**	4.43 ± 2.46	4.11 ± 2.75
Systemic Hypertension	16 (57.1)			
Dyslipidemia	12 (42.9)			
Prostatic hypertrophy	5 (17.9)			
Diabetes	5 (17.9)			
Anxiety	2 (7.1)	**Inferior**		
Atrial fibrillation	2 (7.1)			
Acute myocardial infarction	2 (7.1)			
Stroke/TIA	2 (7.1)			
Arthrosis	2 (7.1)			
Celiac disease	1 (3.6)	**Superior**		
Dilated cardiomyopathy	1 (3.6)			
Depression	1 (3.6)			
Hyperuricemia	1 (3.6)			
Migraine	1 (3.6)			
Osteoporosis	1 (3.6)			
Psoriatic arthritis	1 (3.6)			
RCU	1 (3.6)			
**Topical therapy**				
Tafluprost	17 (42.5)			
Latanoprost	12 (30)			
Bimatoprost	6 (15)			
Travoprost	5 (12.5)			

**Table 3:** Comparisons of the pattern electroretinogram variations over the time depending on the intake of CIT/HOMO/B3/PPQ or CIT800.

**Parameter**	**Pre** **Mean ± SD** **(95%CI)**	**Post** **Mean ± SD** **(95%CI)**	**Meandiff (95%CI)**	***p***	**Pre** **Mean ± SD** **(95%CI)**	**Post** **Mean ± SD** **(95%CI)**	**Meandiff (95%CI)**	***p***
**Pattern electroretinogram (PERG) -transient PERG**	**CIT800**				**CIT/HOMO/B3/PQQ**			
**Central**								
Amplitude Amp-p50 (μV)	5.67 ± 2.86 (4.75 to 6.59)	6.57 ± 2.73 (5.70 to 7.45)	−0.90 (−2.15 to 0.34)	0.15	5.59 ± 2.68 (4.74 to 6.45)	6.63 ± 3.00 (5.67 to 7.59)	−1.04 (−2.31 to 0.23)	0.11
Latency Lat-p50 (ms)	68.24 ± 5.44 (66.50 to 69.98)	66.69 ± 4.76 (65.17 to 68.22)	1.55 (−0.73 to 3.82)	0.18	67.90 ± 4.67 (66.41 to 69.40)	64.56 ± 3.62 (63.40 to 65.72)	3.34 (1.48 to 5.20)	**0.0006**
Amplitude Amp-n95 (μV)	7.85 ± 3.59 (6.70 to 9.00)	9.51 ± 4.06 (8.21 to 10.81)	−1.66 (−3.37 to 0.05)	0.06	8.65 ± 4.55 (7.20 to 10.10)	8.93 ± 4.06 (7.63 to 10.23)	−0.28 (−2.20 to 1.64)	0.77
Latency Lat-n95 (ms)	118.72 ± 8.36 (116.04 to 121.39)	113.85 ± 8.64 (111.08 to 116.61)	4.87 (1.08 to 8.65)	**0.01**	118.24 ± 10.13 (115.00 to 121.48)	114.60 ± 8.62 (111.85 to 117.36)	3.64 (−0.55 to 7.83)	0.09
**Inferior**								
Amplitude Amp-p50 (μV)	3.01 ± 1.56 (2.51 to 3.51)	4.32 ± 1.79 (3.75 to 4.89)	−1.31 (−2.05 to −0.56)	**0.0008**	3.20 ± 1.51 (2.72 to 3.68)	4.24 ± 1.81 (3.66 to 4.82)	−1.04 (−1.78 to −0.30)	**0.006**
Latency Lat-p50 (ms)	66.76 ± 5.63 (64.96 to 68.56)	65.70 ± 4.24 (64.35 to 67.06)	1.06 (−1.16 to 3.28)	0.34	66.23 ± 4.87 (64.67 to 67.78)	63.58 ± 2.93 (62.65 to 64.52)	2.65 (0.85 to 4.43)	**0.004**
Amplitude Amp-n95 (μV)	4.48 ± 2.53 (3.67 to 5.29)	5.69 ± 2.78 (4.80 to 6.58)	−1.21 (−2.39 to −0.03)	**0.04**	4.52 ± 2.43 (3.74 to 5.29)	5.69 ± 2.79 (4.80 to 6.58)	−1.17 (−2.34 to −0.01)	**0.048**
Latency Lat-n95 (ms)	118.95 ± 10.32 (115.65 to 122.25)	112.08 ± 10.51 (108.72 to 115.44)	6.87 (2.23 to 11.51)	**0.004**	115.64 ± 11.05 (112.11 to 119.17)	111.99 ± 9.54 (108.94 to 115.04)	3.65 (−0.95 to 8.24)	0.12
**Superior**								
Amplitude Amp-p50 (μV)	3.71 ± 1.88 (3.10 to 4.31)	4.72 ± 2.16 (4.03 to 5.41)	−1.01 (−1.92 to −0.11)	**0.03**	3.81 ± 1.99 (3.17 to 4.44)	5.05 ± 2.62 (4.22 to 5.89)	−1.24 (−2.28 to −0.21)	**0.02**
Latency Lat-p50 (ms)	67.34 ± 5.52 (65.58 to 69.11)	67.48 ± 6.34 (65.45 to 69.51)	−0.14 (−2.78 to 2.51)	0.92	69.71 ± 6.98 (67.48 to 71.95)	65.03 ± 5.00 (63.43 to 66.63)	4.68 (1.98 to 7.39)	**0.0009**
Amplitude Amp-n95 (μV)	5.55 ± 2.83 (4.65 to 6.46)	5.96 ± 2.69 (5.10 to 6.82)	−0.41 (−1.63 to 0.82)	0.51	5.12 ± 2.53 (4.31 to 5.93)	7.10 ± 3.58 (5.95 to 8.25)	−1.98 (−3.37 to −0.60)	**0.006**
Latency Lat-n95 (ms)	112.40 ± 25.86 (104.13 to 120.67)	110.37 ± 11.11 (106.82 to 113.93)	2.03 (−6.83 to 10.89)	0.65	111.88 ± 19.95 (105.50 to 118.26)	110.95 ± 9.48 (107.91 to 113.98)	0.93 (−6.06 to 7.93)	0.79

**Table 4:** Cross-over analysis of the pattern ERG.

Parameter	Mean ± SD (95%CI)	Mean ± SD (95%CI)	Meandiff (95%CI)	*p*
**Pattern ERG Central**				
Amplitude Amp-p50 (μV)				
Cebrolux vs. Neuprozin	6.12 ± 2.82 (5.49 to 6.75)	6.11 ± 2.88 (5.47 to 6.75)	0.01 (−0.31 to 0.33)	0.96
Cebrolux/Neuprozin vs. Neuprozin/Cebrolux	6.15 ± 2.95 (5.49 to 6.81)	6.08 ± 2.73 (5.48 to 6.69)	0.07 (−1.15 to 1.28)	0.91
Latency Lat-p50 (ms)				
Cebrolux vs. Neuprozin	67.47 ± 5.14 (66.32 to 68.61)	66.23 ± 4.48 (65.23 to 67.23)	1.24 (0.37 to 2.10)	**0.006**
Cebrolux/Neuprozin vs. Neuprozin/Cebrolux	66.40 ± 4.95 (65.30 to 67.50)	67.30 ± 4.73 (66.25 to 68.35)	−0.90 (−2.79 to 0.99)	0.35
Amplitude Amp-n95 (μV)				
Cebrolux vs. Neuprozin	8.68 ± 3.90 (7.81 to 9.55)	8.79 ± 4.29 (7.84 to 9.74)	−0.11 (−0.39 to 0.17)	0.44
Cebrolux/Neuprozin vs. Neuprozin/Cebrolux	8.39 ± 3.85 (7.54 to 9.25)	9.08 ± 4.31 (8.12 to 10.04)	−0.69 (−2.48 to 1.10)	0.45
Latency Lat-n95 (ms)				
Cebrolux vs. Neuprozin	116.28 ± 8.79 (114.32 to 118.24)	116.42 ± 9.52 (114.3 to 118.54)	−0.14 (−1.60 to 1.31)	0.85
Cebrolux/Neuprozin vs. Neuprozin/Cebrolux	116.66 ± 8.69 (114.73 to 118.59)	116.04 ± 9.61 (113.9 to 118.18)	0.62 (−3.10 to 4.33)	0.74
**Pattern ERG Inferior**				
Amplitude Amp-p50 (μV)				
Cebrolux vs. Neuprozin	3.67 ± 1.79 (3.27 to 4.07)	3.72 ± 1.74 (3.33 to 4.11)	−0.05 (−0.27 to 0.16)	0.61
Cebrolux/Neuprozin vs. Neuprozin/Cebrolux	3.63 ± 1.79 (3.23 to 4.03)	3.76 ± 1.74 (3.37 to 4.15)	−0.13 (−0.84 to 0.58)	0.71
Latency Lat-p50 (ms)				
Cebrolux vs. Neuprozin	66.23 ± 4.48 (65.13 to 67.34)	64.91 ± 4.21 (63.97 to 65.84)	1.32 (0.44 to 2.22)	**0.004**
Cebrolux/Neuprozin vs. Neuprozin/Cebrolux	65.17 ± 4.74 (64.12 to 66.23)	65.97 ± 4.54 (64.95 to 66.98)	−0.80 (−2.60 to 1.02)	0.39
Amplitude Amp-n95 (μV)				
Cebrolux vs. Neuprozin	5.09 ± 2.71 (4.48 to 5.69)	5.10 ± 2.66 (4.51 to 5.70)	−0.01 (−0.23 to 0.19)	0.86
Cebrolux/Neuprozin vs. Neuprozin/Cebrolux	5.09 ± 2.71 (4.48 to 5.69)	5.11 ± 2.66 (4.51 to 5.7)	−0.02 (−1.17 to 1.13)	0.97
Latency Lat-n95 (ms)				
Cebrolux vs. Neuprozin	115.52 ± 10.91 (113.09 to 117.95)	113.82 ± 10.42 (111.5 to 116.14)	1.70 (0.09 to 3.31)	**0.038**
Cebrolux/Neuprozin vs. Neuprozin/Cebrolux	115.47 ± 10.48 (113.14 to 117.80)	113.86 ± 10.86 (111.44 to 116.28)	1.61 (−2.71 to 5.94)	0.46
**Pattern ERG Superior**				
Amplitude Amp-p50 (μV)				
Cebrolux vs. Neuprozin	4.21 ± 2.08 (3.75 to 4.68)	4.43 ± 2.39 (3.90 to 4.96)	−0.22 (−0.53 to 0.10)	0.17
Cebrolux/Neuprozin vs. Neuprozin/Cebrolux	4.38 ± 2.37 (3.85 to 4.91)	4.26 ± 2.11 (3.79 to 4.73)	0.12 (−0.80 to 1.04)	0.81
Latency Lat-p50 (ms)				
Cebrolux vs. Neuprozin	67.41 ± 5.91 (66.1 to 68.73)	67.37 ± 6.48 (65.93 to 68.81)	0.04 (−0.66 to 0.75)	0.91
Cebrolux/Neuprozin vs. Neuprozin/Cebrolux	66.19 ± 5.36 (64.99 to 67.38)	68.6 ± 6.72 (67.1 to 70.09)	−2.41 (−4.99 to 0.17)	0.07
Amplitude Amp-n95 (μV)				
Cebrolux vs. Neuprozin	5.76 ± 2.75 (5.14 to 6.37)	6.11 ± 3.24 (5.39 to 6.83)	−0.35 (−0.60 to −0.10)	**0.006**
Cebrolux/Neuprozin vs. Neuprozin/Cebrolux	6.33 ± 3.30 (5.59 to 7.06)	5.54 ± 2.63 (4.95 to 6.12)	0.79 (−0.49 to 2.07)	0.22
Latency Lat-n95 (ms)				
Cebrolux vs. Neuprozin	111.39 ± 19.8 (106.98 to 115.79)	111.41 ± 15.53 (107.96 to 114.87)	−0.02 (−4.91 to 4.85)	0.99
Cebrolux/Neuprozin vs. Neuprozin/Cebrolux	111.67 ± 19.36 (107.36 to 115.98)	111.13 ± 16.06 (107.55 to 114.7)	0.54 (−5.74 to 6.84)	0.86

**Table 5:** Comparisons of the visual acuity (VA), intraocular pressure (IOP), visual field mean deviation (MD), and visual field pattern standard deviation (PSD) over the time depending on the intake of CIT/HOMO/B3/PPQ or CIT800.

Parameter	Pre Mean ± SD (95%CI)	Post Mean ± SD (95%CI)	Meandiff (95%CI)	*p*	Pre Mean ± SD (95%CI)	Post Mean ± SD (95%CI)	Meandiff (95%CI)	*p*
	CIT800				CIT/HOMO/PQQ			
Visual acuity (decimals)	9.42 ± 1.46 (8.96 to 9.89)	8.47 ± 2.00 (7.83 to 9.11)	0.95 (0.17 to 0.73)	**0.02**	8.53 ± 2.01 (7.88 to 9.17)	9.43 ± 1.47 (8.96 to 9.89)	−0.90 (−1.68 to −0.11)	**0.02**
IOP (mmHg)	12.32 ± 1.98 (11.69 to 12.96)	12.65 ± 2.20 (11.95 to 13.35)	−0.32 (−1.26 to 0.61)	0.49	12.85 ± 2.33 (12.11 to 13.59)	12.38 ± 1.97 (11.74 to 13.01)	0.47 (−0.48 to 1.43)	0.33
MD (dB)	−2.10 ± 1.63 (−2.62 to −1.58)	−2.69 ± 2.73 (−3.56 to −1.81)	0.58 (−0.42 to 1.59)	0.25	−3.01 ± 1.68 (−3.55 to −2.47)	−1.77 ± 2.03 (−2.42 to −1.12)	−1.24 (−2.07 to −0.41)	**0.004**
PSD (dB)	2.42 ± 1.23 (2.03 to 2.82)	2.27 ± 2.01 (1.63 to 2.92)	0.15 (−0.59 to 0.89)	0.69	2.90 ± 1.27 (2.49 to 3.30)	2.14 ± 1.52 (1.66 to 2.63)	0.76 (0.13 to 1.37)	**0.02**

**Table 6:** Cross over analysis for the visual acuity, the intraocular pressure (IOP), and the visual field (mean deviation, MD and pattern standard deviation, PSD).

Parameter	Mean ± SD (95%CI)	Mean ± SD (95%CI)	Meandiff (95%CI)	*p*
Visual acuity (decimals, Snellen)				
Cebrolux vs. Neuprozin	8.95 ± 1.81 (8.55 to 9.35)	8.98 ± 1.81 (8.57 to 9.38)	−0.03 (−0.07 to 0.02)	0.32
Cebrolux/Neuprozin vs. Neuprozin/Cebrolux	9.43 ± 1.46 (9.1 to 9.75)	8.5 ± 1.99 (8.06 to 8.94)	0.93 (0.14 to 1.70)	0.02
IOP (mmHg)				
Cebrolux vs. Neuprozin	12.49 ± 2.09 (12.02 to 12.95)	12.61 ± 2.16 (12.13 to 13.09)	−0.12 (−0.54 to 0.29)	0.55
Cebrolux/Neuprozin vs. Neuprozin/Cebrolux	12.35 ± 1.96 (11.91 to 12.79)	12.75 ± 2.25 (12.25 to 13.25)	−0.40 (−1.25 to 0.45)	0.35
MD (decibels)				
Cebrolux vs. Neuprozin	−2.39 ± 2.25 (−2.90 to −1.89)	−2.39 ± 1.95 (−2.82 to −1.95)	0.00 (−0.48 to 0.47)	0.98
Cebrolux/Neuprozin vs. Neuprozin/Cebrolux	−1.93 ± 1.84 (−2.34 to −1.53)	−2.85 ± 2.26 (−3.35 to −2.35)	0.92 (0.12 to 0.70)	0.02
PSD (dB)				
Cebrolux vs. Neuprozin	2.35 ± 1.66 (1.98 to 2.72)	2.52 ± 1.44 (2.2 to 2.84)	−0.17 (−0.59 to 0.25)	0.43
Cebrolux/Neuprozin vs. Neuprozin/Cebrolux	2.28 ± 1.38 (1.98 to 2.59)	2.58 ± 1.70 (2.21 to 2.96)	−0.30 (−0.84 to 0.24)	0.27

**Table 7:** Comparisons of the quality of life examined with National Eye Institute Visual Function Questionnaire 25-item (NEI-VFQ 25) variations over the time depending on the intake of CIT/HOMO/B3/PPQ or CIT800.

Parameter	Pre Mean ± SD (95%CI)	Post Mean ± SD (95%CI)	Meandiff (95%CI)	*p*	Pre Mean ± SD (95%CI)	Post Mean ± SD (95%CI)	Meandiff (95%CI)	*p*
Scale	CIT800				CIT/HOMO/B3/PQQ			
General Health—GH	74.75 ± 20.28 (65.26 to 84.24)	83.28 ± 8.57 (79.26 to 87.29)	−8.53 (−18.65 to 1.60)	0.09	79.13 ± 8.86 (74.98 to 83.27)	76.75 ± 11.44 (71.39 to 82.11)	2.38 (−4.18 to 8.93)	0.47
General Vision—GV	80.5 ± 11.99 (74.89 to 86.11)	85.55 ± 6.56 (82.48 to 88.62)	−5.05 (−11.30 to 1.20)	0.11	80.13 ± 6.2 (77.22 to 83.03)	76.75 ± 11.44 (71.39 to 82.11)	−1.75 (−5.68 to 2.18)	0.37
Ocular Pain—OP	88.13 ± 11.09 (82.94 to 93.31)	89.38 ± 9.31 (85.02 to 93.73)	−1.25 (−7.80 to 5.30)	0.7	87.78 ± 9.06 (83.53 to 92.02)	87.5 ± 11.47 (82.13 to 92.87)	0.28 (−6.34 to 6.89)	0.93
Near Activities—NA	92.92 ± 6.91 (89.68 to 96.15)	94.15 ± 5.37 (91.63 to 96.66)	−1.23 (−5.19 to 2.73)	0.53	91.09 ± 8.03 (87.34 to 94.85)	90 ± 9.97 (85.33 to 94.67)	1.09 (−4.70 to 6.89)	0.71
Distance Activities—DADistance Activities—DA	89.58 ± 6.55 (86.52 to 92.65)	91.04 ± 6.24 (88.12 to 93.96)	−1.46 (−5.55 to 2.64)	0.47	87.26 ± 7.3 (83.84 to 90.67)	87.72 ± 9.4 (83.32 to 92.11)	−0.46 (−5.84 to 4.93)	0.86
Social Functioning—SF	94.58 ± 6.21 (91.68 to 97.49)	95.42 ± 5.72 (92.74 to 98.09)	−0.84 (−4.65 to 2.99)	0.66	87.33 ± 5.99 (84.53 to 90.13)	87.08 ± 8.75 (82.99 to 91.18)	0.25 (−4.55 to 5.05)	0.92
Mental Health—MH	90.5 ± 6.05 (87.67 to 93.33)	90.5 ± 6.05 (87.67 to 93.33)	0.00 (−3.87 to 3.87)	1	90.19 ± 6.44 (87.17 to 93.2)	89.69 ± 8.03 (85.93 to 93.44)	0.50 (−4.16 to 5.16)	0.83
Role Dependency—RD	94.38 ± 7.00 (91.1 to 97.65)	95 ± 6.28 (92.06 to 97.94)	−0.62 (−4.88 to 3.63)	0.77	88.58 ± 8.61 (84.55 to 92.61)	87.5 ± 9.72 (82.95 to 92.05)	1.08 (−4.80 to 6.96)	0.71
Vision-Specific Dependency—VSD	92.19 ± 8.81 (88.06 to 96.31)	94.13 ± 6.5 (91.08 to 97.17)	−1.94 (−6.89 to 3.02)	0.43	90.83 ± 7.5 (87.32 to 94.34)	91.44 ± 9.47 (87.01 to 95.87)	−0.61 (−6.08 to 4.86)	0.82
Driving—D	96.67 ± 4.19 (94.71 to 98.63)	97.08 ± 4.08 (95.17 to 98.99)	−0.41 (−3.06 to 2.23)	0.75	91.85 ± 8.29 (87.97 to 95.73)	90.92 ± 11.19 (85.68 to 96.16)	0.93 (−5.37 to 7.24)	0.77
Color Vision—CV	93.75 ± 13.75 (87.31 to 100.19)	96.3 ± 8.16 (92.48 to 100.12)	−2.55 (−9.84 to 4.74)	0.48	94.05 ± 9.47 (89.62 to 98.48)	90 ± 14.96 (83 to 97)	4.05 (−3.96 to 12.06)	0.31
Peripheral Vision—PV	87.5 ± 15.17 (80.4 to 94.6)	90.95 ± 11.14 (85.74 to 96.16)	3.45 (−11.97 to 5.07)	0.42	91.55 ± 9.32 (87.19 to 95.91)	85 ± 14.96 (78 to 92)	6.55 (−1.48 to 14.58)	0.11
Total mean	90.61 ± 4.93 (88.3 to 92.92)	92.92 ± 4.32 (90.9 to 94.94)	2.31 (−5.28 to 0.65)	0.12	90.71 ± 3.7 (88.98 to 92.44)	88.02 ± 6.28 (85.08 to 90.96)	2.69 (−0.64 to 6.02)	0.11

**Table 8:** Cross over analysis of the quality of life, examined by 25-item National Eye Institute Visual Function Questionnaire (NEI-VFQ 25).

Parameter	Mean ± SD (95%CI)	Mean ± SD (95%CI)	Meandiff (95%CI)	*p*
General health—GH				
Cebrolux vs. Neuprozin	75.75 ± 16.28 (70.54 to 80.96)	81.20 ± 8.86 (78.37 to 84.03)	−5.45 (−9.57 to −1.34)	**0.01**
Cebrolux/Neuprozin vs. Neuprozin/Cebrolux	79.01 ± 15.96 (73.91 to 84.12)	77.94 ± 10.17 (74.68 to 81.19)	1.07 (−6.29 to 8.44)	0.77
General vision—GV				
Cebrolux vs. Neuprozin	81.19 ± 9.41 (78.18 to 84.20)	82.84 ± 6.87 (80.64 to 85.04)	−1.65 (−3.91 to 0.61)	0.15
Cebrolux/Neuprozin vs. Neuprozin/Cebrolux	83.03 ± 9.88 (79.87 to 86.18)	81.00 ± 6.12 (79.04 to 82.96)	2.02 (−2.64 to 6.69)	0.39
Ocular pain—OP				
Cebrolux vs. Neuprozin	87.81 ± 11.14 (84.25 to 91.38)	88.58 ± 9.11 (85.66 to 91.49)	−0.77 (−2.33 to 0.81)	0.33
Cebrolux/Neuprozin vs. Neuprozin/Cebrolux	88.75 ± 10.13 (85.51 to 91.99)	87.64 ± 10.21 (84.37 to 90.90)	1.11 (−5.28 to 7.51)	0.73
Near activities—NA				
Cebrolux vs. Neuprozin	91.46 ± 8.59 (88.71 to 94.21)	92.62 ± 6.91 (90.41 to 94.83)	−1.16 (−2.47 to 0.15)	0.08
Cebrolux/Neuprozin vs. Neuprozin/Cebrolux	93.53 ± 6.14 (91.57 to 95.49)	90.55 ± 8.95 (87.68 to 93.41)	2.99 (−1.80 to 7.77)	0.21
Distance activities—DA				
Cebrolux vs. Neuprozin	88.65 ± 8.05 (86.07 to 91.23)	89.15 ± 6.97 (86.92 to 91.38)	−0.50 (−1.91 to 0.91)	0.48
Cebrolux/Neuprozin vs. Neuprozin/Cebrolux	90.31 ± 6.36 (88.28 to 92.35)	87.49 ± 8.31 (84.83 to 90.14)	2.82 (−1.75 to 7.40)	0.22
Vision specific social functioning—VSSF				
Cebrolux vs. Neuprozin	90.83 ± 8.40 (88.15 to 93.52)	91.37 ± 7.08 (89.11 to 93.64)	−0.54 (−1.70 to 0.62)	0.35
Cebrolux/Neuprozin vs. Neuprozin/Cebrolux	95.00 ± 5.91 (93.11 to 96.89)	87.21 ± 7.40 (84.84 to 89.57)	7.79 (3.61 to 11.97)	0.0005
Vision specific mental health—MH				
Cebrolux vs. Neuprozin	90.09 ± 7.03 (87.85 to 92.34)	90.34 ± 6.17 (88.37 to 92.32)	−0.25 (−1.85 to 1.35)	0.75
Cebrolux/Neuprozin vs. Neuprozin/Cebrolux	90.50 ± 5.97 (88.59 to 92.41)	89.94 ± 7.19 (87.64 to 92.24)	0.56 (−3.41 to 4.54)	0.78
Vision specific role dependency—RD				
Cebrolux vs. Neuprozin	90.94 ± 9.06 (88.04 to 93.83)	91.79 ± 8.12 (89.19 to 94.39)	−0.85 (−2.15 to 0.45)	0.19
Cebrolux/Neuprozin vs. Neuprozin/Cebrolux	94.69 ± 6.57 (92.59 to 96.79)	88.04 ± 9.08 (85.14 to 90.94)	6.65 (1.68 to 11.61)	0.01
Vision specific dependency—VSD				
Cebrolux vs. Neuprozin	91.81 ± 9.04 (88.92 to 94.70)	92.48 ± 7.13 (90.20 to 94.76)	−0.67 (−2.27 to 0.94)	0.41
Cebrolux/Neuprozin vs. Neuprozin/Cebrolux	93.16 ± 7.71 (90.69 to 95.62)	91.13 ± 8.44 (88.44 to 93.83)	2.02 (−2.94 to 6.99)	0.41
Driving—D				
Cebrolux vs. Neuprozin	93.79 ± 8.84 (90.97 to 96.62)	94.47 ± 6.97 (92.24 to 96.7)	−0.68 (−2.05 to 0.70)	0.33
Cebrolux/Neuprozin vs. Neuprozin/Cebrolux	96.87 ± 4.09 (95.57 to 98.18)	91.38 ± 9.73 (88.27 to 94.50)	5.49 (0.85 to 10.13)	0.02
Color vision—CV				
Cebrolux vs. Neuprozin	91.88 ± 14.31 (87.30 to 96.45)	95.18 ± 8.80 (92.36 to 97.99)	−3.30 (−5.60 to −1.00)	**0.006**
Cebrolux/Neuprozin vs. Neuprozin/Cebrolux	95.03 ± 11.24 (91.43 to 98.62)	92.03 ± 12.53 (88.02 to 96.03)	3.00 (−4.28 to 10.28)	0.41
Peripheral vision—PV				
Cebrolux vs. Neuprozin	86.25 ± 14.93 (81.48 to 91.02)	91.25 ± 10.14 (88.01 to 94.49)	−5.00 (−7.88 to −2.12)	**0.001**
Cebrolux/Neuprozin vs. Neuprozin/Cebrolux	89.23 ± 13.25 (84.99 to 93.46)	88.28 ± 12.74 (84.20 to 92.35)	0.95 (−6.78 to 8.68)	0.81
Total mean score				
Cebrolux vs. Neuprozin	89.32 ± 5.73 (87.48 to 91.15)	91.82 ± 4.12 (90.50 to 93.14)	−2.50 (−4.17 to −0.83)	**0.004**
Cebrolux/Neuprozin vs. Neuprozin/Cebrolux	91.77 ± 4.72 (90.26 to 93.28)	89.37 ± 5.27 (87.68 to 91.05)	2.40 (−0.26 to 5.06)	0.07

## Data Availability

The data presented in this study are available on request from the corresponding author.
